# Mosses in the Kopački Rit Nature Park, Croatia, as bioindicators of a potential radioactive contamination of the middle Danube River basin

**DOI:** 10.1038/s41598-022-15716-3

**Published:** 2022-07-08

**Authors:** Branko Petrinec, Dinko Babić, Tomislav Meštrović, Tomislav Bogdanović, Marina Popijač, Davor Rašeta

**Affiliations:** 1 Institute for Medical Research and Occupational Health, Ksaverska cesta 2, 10000 Zagreb, Croatia; 2 Public Institution “Nature Park Kopački Rit”, Mali Sakadaš 1, 31327 Kopačevo, Bilje, Croatia; 3 Public Institution “Nature Park Medvednica”, Bliznec 70, 10000 Zagreb, Croatia

**Keywords:** Environmental impact, Environmental monitoring

## Abstract

We studied activity concentrations of radionuclides in the Kopački Rit Nature Park using mosses as bioindicators. This area of intact nature is at the tripoint of Croatia, Hungary, and Serbia, being located basically at the centre of the middle Danube River basin. Therefore, it can be easily affected by airborne pollution from various locations in the Middle Europe and beyond. The goal of our research was to assess whether the Park could serve as a location where any new radioactive contamination could be sensitively detected, which implied a necessity for low activity concentrations at the present time. Our gamma-ray spectrometry revealed the presence of only one anthropogenic gamma emitter, that is, ^137^Cs. Its activity concentration in the mosses ranged from 0.7 to 13.1 Bq kg^−1^, being low indeed. Another radionuclide in our focus was ^210^Pb. Generally, its elevated concentrations may signify ecologically undesirable human activities that involve naturally occurring radioactive matter. The activity concentration of ^210^Pb in the mosses was in the range from 183 to 690 Bq kg^−1^. This did not depart from the results of other similar studies and was again low enough for a detection of possible excess amounts of this radionuclide in the future.

## Introduction

Ecological concerns are nowadays among central topics related to the sustainable development of our civilisation. This especially holds true for densely populated areas, where even a modest pollution may pose a public health issue. The source of such a pollution does not have to be close to the polluted area, since many pollutants can travel long distances as part of air masses. In particular, this is not uncommon in case of a leakage of radioactive substances into the environment, as evident from the Chernobyl and Fukushima disasters in 1986 and 2011, respectively^1^. While accidents involving radioactive matter are relatively rare, less dramatic (but still alarming) leakages from nuclear facilities occur from time to time; they may even remain undeclared but sometimes cannot avoid detection due to a global network of monitoring stations^2^. To maintain and even expand this network is a future challenge, and the goal of this paper is to contribute to the related efforts by concentrating on the middle Danube River basin (MDBR), a densely populated area that covers significant part of Middle Europe.

**Figure 1:**
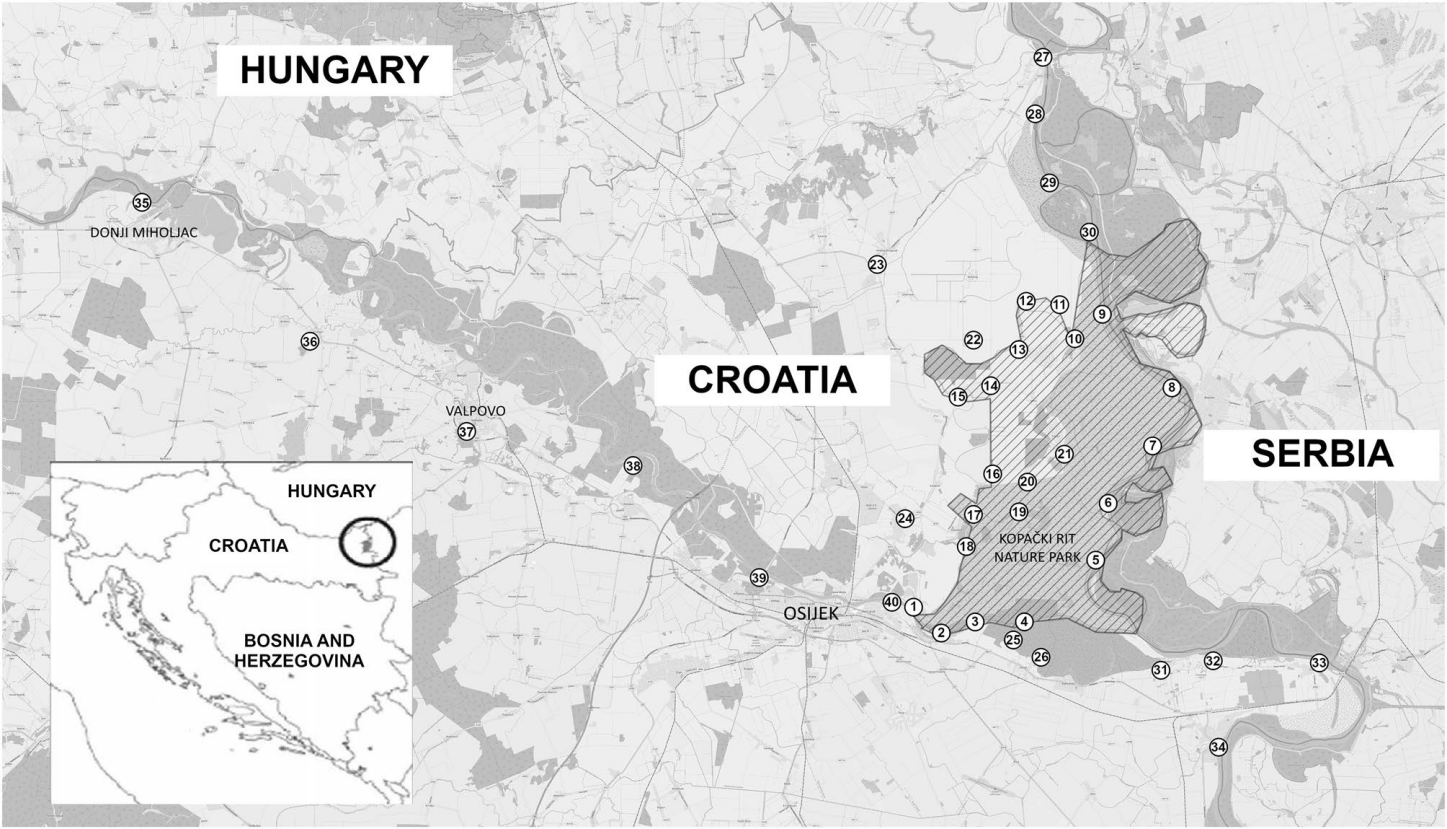
Sampling locations L = 1–40 (encircled numbers) and the position of the studied area on the map (inset). Sampling was carried out inside the KRNP (shaded, L = 1–21), in the vicinity but outside the KRNP (L = 22–26), along the Danube (L = 27–34) and the Drava (IV, L = 35–40).

More precisely, we focus on a possibility of using the Kopački Rit Nature Park (KRNP) in Croatia as a detection site for airborne radioactive pollutants in the MDBR, based on the fact that the KRNP is abundant in suitable bioindicator organisms and, as shown recently^3^, radiologically unpolluted. These properties make the KRNP sensitive to the presence of anthropogenic radionuclides as well as to elevated concentrations of naturally occurring radionuclides, which may appear in consequence of human activities. As shown in Fig. 1, the KRNP is located on the Croatian side of the tripoint of Croatia, Hungary, and Serbia. It comprises 177 km^2^ of mainly intact marshy lowland that contains abundant wildlife and is subject to flooding. It is close to an urban centre (Osijek, ~ 100,000 inhabitants), downstream from a nuclear power plant (Packs, Hungary), in the vicinity of regions of intense agriculture, and bordered by two large rivers (the Danube in the east and the Drava in the south). These are all potential sources of anthropogenic pollution at a local level. Moreover, since the KRNP is about 1200 km from the source of the Danube and 1400 km from the its mouth, i.e., virtually in the centre of the MDBR, airborne radionuclides that would appear over a larger area in the MDBR from a nonlocal source should also be clearly detectable in KRNP.

For any detection of airborne radionuclides, the use of bioindicators is a reasonable choice. The bioindicators in our study were mosses, known for their ability to accumulate toxic or mutagenic substances without suffering substantial damage themselves^4,5^. Mosses absorb matter by the entire surface of the body, through phyllodes, and not only via rhizoids that perform the function of roots, which makes them ideal for studies on airborne pollutants including radionuclides^6^. We collected samples of mosses in the KRNP and its vicinity in order to investigate their current radionuclide content, assess the related overall situation, identify possible hotspots and, if these being absent, prepare data for comparison in case of a future radioactive contamination. Our analytical method was a high-resolution gamma-ray spectrometry, which allowed us to quantify activity concentrations (*A*) of the most significant gamma emitting radionuclides in the environment. Due to its simplicity, sensitivity, and accuracy, this method is the most widely used in studies on environmental radioactivity.

Of anthropogenic radionuclides, we concentrated on ^137^Cs (with a half-live *T*_1/2_ = 30.1 years)—which was actually the only manmade gamma emitter that we could have detected—and the measured activity concentrations set the background values for the monitoring of airborne ^137^Cs in the future. Human activities can result in numerous radionuclides, some of which are gamma emitters and some are not, but radiocaesium is very frequently in the focus of ecological concerns. ^137^Cs is a strong gamma emitter—therefore easily detectable—that can become airborne if being released into the atmosphere by a nuclear explosion or an accident in a nuclear power plant. Another widely studied isotope of caesium is ^134^Cs (*T*_1/2_ = 2.06 years), which was not detected in our study. It is also a strong gamma emitter but can appear in the environment only by a leakage from a nuclear reactor and not due to a nuclear explosion. Elaborated studies of causes and consequences of an anthropogenic radioactive pollution requires detailed analyses of other radionuclides as well, but ^137^Cs and ^134^Cs provide a bulk of relevant information if the fission of uranium is behind a radionuclide release. The abundance of nuclear facilities in Europe clearly calls for a constant awareness based on continual measurements in selected areas, and the research presented in this paper identifies the KRNP as a suitable one for this purpose.

Not every radioecological threat is related to a nuclear facility; some are caused by industrial process which enhance environmental concentrations of naturally occurring radionuclides. In our assessment of this aspect of radioactive pollution, we focused on ^210^Pb (*T*_1/2_ = 22.4 years), since it is a product of the decay of airborne ^222^Rn and is therefore abundant in the air. The obtained reference values are also of importance, but in this case more locally. ^210^Pb is deposited onto the surface of plants due to the deposition of airborne progenies of gaseous ^222^Rn^7^, which makes its concentration in mosses high enough to be measured. Elevated concentrations of ^210^Pb in the air may be related to different human activities, such as the use of soil amendments rich in ^226^Ra (the mother nucleus of ^222^Rn)^8^, burning of coal in power plants and other uses of fossil fuels^9^, etc. Hence, the *A* of ^210^Pb in mosses is also a valuable indicator of human impact on the environment.

## Materials and methods

Altogether 40 samples of mosses were collected at the locations that are in Fig. 1 shown by encircled numbers. In order to obtain more complete information, mosses were sampled inside the KRNP (henceforth, denoted as I; shaded, L = 1–21), in the vicinity but outside the KRNP (II; L = 22–26), along the Danube (III; L = 27–34) and the Drava (IV; L = 35–40). In order to cover the diversity of the KRNP, sampling locations therein were selected by considering different parameters like types of microhabitats and substrates (stone, earth, wood) and the level of anthropogenic influence. Furthermore, we selected the terrain for sampling by taking into account that mosses take up matter primarily from the air, precipitation, soil moisture, surface reservoirs, and floodwater. Hence, sampling was carried out in the areas of occasional floods (henceforth, denoted as Ia; L = 1–5, 7, 8, 16, 18, 20), the edges of Ia (Ib; L = 6, 9, 17, 19, 21), and dry areas (Ic; L = 10–15). The most abundant moss in the KRNP is *Anomodon viticulosus*, and we therefore chose to base our study on it.

The sampling was carried out on several occasions from May through August 2018. Samples were thoroughly cleaned, dried to constant mass, tightly sealed into Marinelli beakers (volume 1 L), and left to rest for about 30 days in order to ensure the establishment of secular equilibria within the ^232^Th and ^238^U decay chains. Therefore, our results for *A* refer to dry mass.

*A* was determined by means of high-resolution γ-ray spectrometry, using a high-purity Ge coaxial detector (Ortec GMX; relative efficiency of 74.3% and energy resolution of 2.23 keV, all at ^60^Co 1.33 MeV). The measurement time was about 80,000 s. The energy and efficiency calibration of the system (40–2000 keV) was carried out using a certified calibration source obtained from the Czech Metrology Institute. All quality assurance procedures in γ-ray spectrometry^10^ were applied, and the method has been accredited according to the ISO 17,025 standard. Our focus was, as outlined above, on ^137^Cs and ^210^Pb, and their activities were quantified from the peaks at 661.7 keV and 46.5 keV, respectively. For completeness, we also addressed other representative naturally occurring radionuclides, that is, ^40^K (peak at 1460.8 keV), ^238^U (in equilibrium with ^234^Th, peak at 63.3 keV and doublet at 92.4–92.8 keV), ^226^Ra (in equilibrium with ^214^Pb, peaks at 295.2 keV and 351.9 keV), and ^232^Th (in equilibrium with ^228^Ac, peaks at 338.3 keV and 911.2 keV), and ^7^Be (peak at 477.6 keV). Since a measurement would be carried out within a month after the corresponding sampling, time correction for radioactive decay was calculated only for ^7^Be (*T*_1/2_ = 53.4 days), as it was negligible for other studied radionuclides due to their long *T*_1/2_.

## Results

Our analysis of the recorded gamma-ray spectra revealed which gamma emitters were present in the samples. We detected ^40^K, ^7^Be, radionuclides from the ^232^Th and ^238^U decay chains, and ^137^Cs as the only anthropogenic radionuclide. Regarding ^40^K, this primordial radionuclide—found in all living organism due to the biogenic nature of potassium—in our samples had *A* between 170 and 450 Bq kg^−1^, the average being 290 Bq kg^−1^. The *A* of cosmogenic ^7^Be (*T*_1/2_ = 50.4 days) was, at the time of the sampling, in the range 80–1050 Bq kg^−1^, averaging at 480 Bq kg^−1^. Since ^7^Be descends towards Earth from the upper atmosphere, its concentration in mosses depends on atmospheric conditions significantly. ^232^Th, ^238^U, and ^226^Ra, as significant representatives of the decay chains before the appearance of radon, were detected in some of our samples but not in all of them, and their maximum values of *A* were 31, 44, and 28 Bq kg^−1^, respectively. The absence of these radionuclides in some samples and their comparatively low activity concentrations in the mosses were related primarily to the fact that their concentrations in the air are generally low.

Activity concentrations of the two radionuclides that are in the focus of this paper, namely ^137^Cs and ^210^Pb, are shown in Tables 1 and 2, respectively, together with the ranges of measured values, arithmetic means (AM), and standard deviations (SD) for sampling areas I-IV. These radionuclides were detected in all of the samples, which was not surprising because of their abundance in the air. Overall, for all samples, the *A* of ^137^Cs was between 0.7 and 13.1 Bq kg^−1^, with AM = 5.3 Bq kg^−1^ and SD = 3.7 Bq kg^−1^. The *A* of ^210^Pb was much higher, in the range 183–690 Bq kg^−1^, with AM = 360 Bq kg^−1^ and SD = 125 Bq kg^−1^. This difference is not surprising, since ^210^Pb is a progeny of gaseous ^222^Rn that constantly emanates into the air due to the decay of naturally occurring ^226^Ra.

**Table 1:** Activity concentrations of ^137^Cs in mosses sampled at locations L = 1–40 that are shown in Fig. 1.

L	*A* (Bq kg^−1^)	L	*A* (Bq kg^−1^)	L	*A* (Bq kg^−1^)	L	*A* (Bq kg^−1^)
**I. Range 1.3–12.4; AM = 5.4; SD = 3.4 (Bq kg**^**−1**^**)** **Ia. Range 2.5–12.4; AM = 6.5; SD = 3.9 (Bq kg**^**−1**^**)** **Ib. Range 2.3–9.4; AM = 5.2; SD = 3.0 (Bq kg**^**−1**^**)** **Ic. Range 1.3–7.1; AM = 3.8; SD = 2.3 (Bq kg**^**−1**^**)**							
1(a)	2.6 ± 0.2	2(a)	3.6 ± 0.2	3(a)	11.0 ± 0.3	4(a)	12.4 ± 0.3
5(a)	9.6 ± 0.3	6(b)	9.4 ± 0.4	7(a)	4.1 ± 0.3	8(a)	6.5 ± 0.3
9(b)	3.6 ± 0.2	10(c)	7.1 ± 0.2	11(c)	1.3 ± 0.2	12(c)	1.8 ± 0.2
13(c)	6.1 ± 0.3	14(c)	3.3 ± 0.2	15(c)	2.9 ± 0.3	16(a)	2.6 ± 0.3
17(b)	2.3 ± 0.2	18(a)	10.1 ± 0.3	19(b)	7.3 ± 0.3	20(a)	2.5 ± 0.2
21(b)	3.3 ± 0.3						
**II. Range 0.7–10.0 ; AM = 4.4; SD = 3.8 (Bq kg**^**−1**^**)**							
22	3.8 ± 0.2	23	0.7 ± 0.1	24	10.0 ± 0.3	25	6.2 ± 0.2
26	1.3 ± 0.1						
**III. Range 0.9–13.1; AM = 7.8; SD = 4.5 (Bq kg**^**−1**^**)**							
27	13.1 ± 0.5	28	6.7 ± 0.3	29	11.7 ± 0.3	30	12.7 ± 0.3
31	4.3 ± 0.3	32	0.9 ± 0.1	33	8.3 ± 0.4	34	4.5 ± 0.2
**IV. Range 1.0–3.9; AM = 2.5; SD = 1.1 (Bq kg**^**−1**^**)**							
35	1.0 ± 0.1	36	1.9 ± 0.2	37	2.0 ± 0.2	38	3.3 ± 0.2
39	3.1 ± 0.2	40	3.9 ± 0.2				

Results are presented in Bq per kg of dry mass, with uncertainties expressed using the coverage factor two. For area I, letters in brackets next to location numbers indicate the type (Ia, Ib, Ic) of terrain. Ranges of measured values, AM, and SD are listed for sampling areas I-IV and also for terrain types Ia, Ib, and Ic. If all samples are treated as a single dataset, the range of values is 0.7–13.1 Bq kg^−1^, whereas AM = 5.3 Bq kg^−1^ and SD = 3.7 Bq kg^−1^.

**Table 2:** Activity concentrations of ^210^Pb in mosses sampled at locations L = 1–40 that are shown in Fig. 1.

L	*A* (Bq kg^−1^)	L	*A* (Bq kg^−1^)	L	*A* (Bq kg^−1^)	L	*A* (Bq kg^−1^)
**I. Range 220–600; AM = 370; SD = 110 (Bq kg**^**-1**^**)** **Ia. Range 220–520; AM = 320; SD = 90 (Bq kg**^**-1**^**)** **Ib. Range 220–520; AM = 360; SD = 120 (Bq kg**^**-1**^**)** **Ic. Range 390–600; AM = 465; SD = 80 (Bq kg**^**-1**^**)**							
1(a)	520 ± 10	2(a)	330 ± 10	3(a)	310 ± 10	4(a)	335 ± 9
5(a)	220 ± 10	6(b)	260 ± 10	7(a)	330 ± 10	8(a)	320 ± 10
9(b)	430 ± 10	10(c)	390 ± 10	11(c)	600 ± 10	12(c)	520 ± 10
13(c)	400 ± 10	14(c)	440 ± 10	15(c)	440 ± 10	16(a)	220 ± 10
17(b)	520 ± 10	18(a)	220 ± 10	19(b)	380 ± 10	20(a)	350 ± 10
21(b)	220 ± 10						
**II. Range 183–390; AM = 280; SD = 80 (Bq kg**^**−1**^**)**							
22	390 ± 10	23	183 ± 8	24	332 ± 9	25	271 ± 8
26	229 ± 7						
**III. Range 220–690; AM = 390; SD = 160 (Bq kg**^**−1**^**)**							
27	220 ± 10	28	300 ± 10	29	310 ± 10	30	370 ± 10
31	270 ± 10	32	690 ± 10	33	550 ± 20	34	380 ± 10
**IV. Range 234–660; AM = 380; SD = 160 (Bq kg**^**−1**^**)**							
35	234 ± 9	36	300 ± 10	37	660 ± 10	38	318 ± 9
39	460 ± 10	40	305 ± 8				

Results are presented in Bq per kg of dry mass, with uncertainties expressed using the coverage factor two. For area I, letters in brackets next to location numbers indicate the type (Ia, Ib, Ic) of terrain. Ranges of measured values, AM, and SD are listed for sampling areas I-IV and also for terrain types Ia, Ib, and Ic. If all samples are treated as a single dataset, the range of values is 183–690 Bq kg^−1^, whereas AM = 360 Bq kg^−1^ and SD = 125 Bq kg^−1^.

Regarding similarities and differences among activity concentrations in sampling areas I-IV and terrain types Ia, Ib, and Ic, it is possible to draw certain conclusions from the values of AM and SD, by comparing differences between the values of AM and the widths of the statistical distributions of *A* as represented by SD. A more quantitative insight can be obtained by carrying out the Mann–Whitney two-sample rank-sum test^11^. This method is appropriate for testing the null hypothesis that two sets of data are basically equal. The method relies on calculating the *U* parameter of the test, comparing it with the tabulated values for a given significance level α^12^ and deciding on the validity of the null hypothesis. We carried out the test by comparing pairs of datasets in Tables 1 and 2 at a usual significance level α = 0.05, two-tailed test, and the results are summarised in Table 3. A significant difference was found in only 4 out of 36 cases.

**Table 3:** Summary of the results of the application of the Mann–Whitney test, two-tailed, to sets of data in Tables 1 and 2.

Set 1	Set 2	*n*_1_	*n*_2_	^137^Cs		^210^Pb	
				*U*	Significantly different	*U*	Significantly different
Ia	Ib	10	5	18.5	No	18	No
Ia	Ic	10	6	17	No	5	Yes
Ib	Ic	5	6	8	No	7	No
Ia	II	10	5	16	No	22	No
Ia	III	10	8	29	No	31	No
Ia	IV	10	6	10	Yes	28	No
Ib	II	5	5	11	No	8	No
Ib	III	5	8	12	No	19	No
Ib	IV	5	6	5.5	No	14	No
Ic	II	6	5	14.5	No	0.5	Yes
Ic	III	6	8	11	No	11	No
Ic	IV	6	6	14.5	No	10	No
I	II	21	5	42.5	No	30.5	No
I	III	21	8	52	No	81	No
I	IV	21	6	30	No	58	No
II	III	5	8	10	No	13	No
II	IV	5	6	12	No	9	No
III	IV	8	6	6	Yes	23.5	No

The sizes of the compared sets are *n*_1_ and *n*_2_, and the significance level is α = 0.05.

## Discussion

The suitability of mosses as biomonitors can be related to their relatively primitive structure in comparison to vascular plants, which leads to a slower exchange of matter in their tissues and enhances the accumulation of different elements. Most mosses absorb water, inorganic substances, and minerals mainly through phyllodes—directly from the air, precipitation, soil moisture, surface reservoirs, and floodwater—whereas the absorption from the substrate via rhizoids is less important^13,14^. Due to their strong absorption and retention of airborne substances^15–17^, and in spite of additional pathways of the absorption of matter, mosses have been recognised as reliable biomonitors for atmospheric pollution. This has been used worldwide to monitor environmental stress caused by numerous non-radioactive^18–22^ and radioactive^9,18,22–29^ elements. Since mosses appear in different habitats, they have been for a long time used to record the dynamics of pollution within small areas such as industrial zones^30–32^, cities^14,33^ or motorways^34,35^.

Radioecological concerns are usually focused on anthropogenic radionuclides and excessive amounts of naturally occurring radionuclides due to human activities. Of gamma emitting anthropogenic radionuclides, we detected only ^137^Cs in relatively small concentrations—as can be seen in Table 1. The contemporary appearance of ^137^Cs in nature is primarily a consequence of three contributions: global radioactive fallout originating from nuclear weapons tests, the Chernobyl accident, and the Fukushima accident. The contribution of the Chernobyl accident to the total budget of ^137^Cs in the studied area was, at the time shortly after the release, at the level of the background set by the global fallout, i.e., considerably lower than in other areas at the same distance from Chernobyl^36,37^. This was a consequence of atmospheric factors that are outside the scope of this paper. Although an influx of fission products (^137^Cs, ^134^Cs, and ^131^I) from the Fukushima accident was observed in Croatia^23,38^, its magnitude was comparatively low^39^, and this did not increase the concentration of ^137^Cs in the studied area significantly. Moreover, a recent comprehensive study on the radioactivity of soil in Croatia revealed that the KRNP was situated within a part of the country where the concentrations of ^137^Cs in soil were the lowest, below 5 Bq kg^−1^^40^.

The *A* of ^137^Cs in the mosses studied here was particularly low, which becomes evident when we compare our results with some other published data on ^137^Cs in mosses from regions that are at similar distances from Chernobyl as the KRNP. In the below listing, the data refer to the situation extrapolated to 2018, calculated by taking into account the radioactive decay, original values, and the elapsed time. The *A* of ^137^Cs in mosses from the Plitvice Lakes National Park in Croatia was in the range 25–1460 Bq kg^−1^ (sampled in 2011–2012)^23^, in Armenia 3–300 Bq kg^−1^ (2018–2020)^29^, in Austria 870–10,680 Bq kg^−1^ (2006)^27^, in Greece 0–400 Bq kg^−1^ (2016)^18^, in Turkey 30–360 Bq kg^1^ (2007) ^25^, and in Serbia 6–65 Bq kg^−1^ (2008)^28^.

Hence, a number of combined factors have resulted in comparatively low concentrations of ^137^Cs in mosses from the KRNP. The identified statistical differences between some of the studied sampling areas and terrains, see Table 3, are neither numerous nor drastic, hence they do not change our main conclusion that the KRNP, with its mosses and geographical location, is well suited for a sensitive detection of the appearance of new ^137^Cs in the wider region. This may as well have implications for other radioactive matter that is not naturally occurring. In particular, since no ^134^Cs was detected in our study, the background for its detection is very low (the detection limit being below 0.5 Bq kg^−1^), which implies a high sensitivity to its appearance as a warning to a problem in a facility where the fission of uranium is taking place.

Regarding naturally occurring radionuclides, mosses have been used mostly in studies on ^7^Be^18,28^ and ^210^Pb^9^^,^^18^^,^^22^^,^^23^^,^^28^ due to their abundance in the air. From the radioecological point of view, however, ^7^Be is of no particular interest, because it is just one of cosmogenic radionuclides—the production of which depends solely on the influx of cosmic rays on Earth^41^ and not on any human activity. On the other hand, the impact of ^210^Pb is important in radioecology. Having the longest *T*_1/2_ of the progenies of gaseous ^222^Rn, this radionuclide accumulates on surfaces exposed to the air and remains there for a long time^7^. Excessive amounts of ^210^Pb in components of the environment that are exposed to the air may signal an abundance of airborne ^222^Rn and consequent adverse effects for the health of the exposed population. This could be linked to human activities such as the use of materials rich in ^226^Ra, e.g., some soil amendments used in the contemporary agricultural production. Other sources of excess ^210^Pb in air are industrial processes involving ores that contain significant amounts of ^238^U, and the burning of fossil fuels^9^. Measurements of the *A* of ^210^Pb may serve as a warning system for undesirable effects of these activities on the environment.

That the predominant origin of ^210^Pb in our samples was deposition from the air, and not other mechanisms, can be inferred by comparing results for the *A* of ^226^Ra and of ^210^Pb. The *A* of ^226^Ra was, first of all, in some samples below detection limit (typically, 1 Bq kg^−1^), and its maximum value was 28 Bq kg^−1^. If the origin of ^210^Pb in the samples had been the decay of ^226^Ra taken up by mosses via other pathways, the activities of the two radionuclides would have been similar. However, the measured *A* of ^210^Pb was much higher, implying that the deposition of ^222^Rn and its progenies from the air was the main source of ^210^Pb in our mosses. This leads to a conclusion that mosses in the KRNP can be used as indicators of atmospheric pollution due to the use of materials that contain naturally occurring radioactive matter.

The range of the *A* of ^210^Pb in our samples (183–690 Bq kg^−1^) was similar to that for mosses from the Plitvice Lakes National Park in Croatia, where it was 68–639 Bq kg^−1^^23^. This suggests that it was likely that the origin of ^210^Pb in both areas was the emanation of ^222^Rn from uncultivated soil rather than any human activity. Hence, any significant increase in the *A* of ^210^Pb in mosses from either of the areas would arise suspicion of an anthropogenic pollution. This is especially important for the KRNP, which is surrounded by several potential sources of such a pollution. Ranges of *A* that are not drastically different from the ones for our samples have been found elsewhere too. For instance, 212–532 Bq kg^−1^ in Germany^22^, 147–1920 Bq kg^−1^ in Greece^18^, 526–881 Bq kg^−1^ in Serbia^28^, 199–660 Bq kg^−1^ in Thailand^28^, 220–720 Bq kg^−1^ in Turkey^9^, etc.

## Conclusions

We studied radionuclide activity concentrations in mosses from the Kopački Rit Nature Park in northeastern Croatia, close to the tripoint of Croatia, Hungary, and Serbia. Comprising mainly intact nature and being situated virtually in the centre of the middle Danube River basin, this area is promising for detecting a potential pollution that might originate from a number of sources and affect the entire basin. Mosses, chosen for this study because of their ability to take up airborne radionuclides, were sampled in 2018 and measured for radionuclide content by means of high-resolution gamma-ray spectrometry. We detected radionuclides from the ^232^Th and ^238^U decay chains, ^40^K, ^7^Be, and ^137^Cs. From the point of view of radioecology, the most interesting were ^210^Pb and ^137^Cs, as their concentrations in the air are sensitive to human activities. The activity concentrations of ^137^Cs were particularly low, ranging from 0.7 to 13.1 Bq kg^−1^. Thus low values imply a considerable sensitivity to any extra amount of ^137^Cs that might arise from facilities and events that involve nuclear matter. While an appearance of extra ^137^Cs could originate from a faraway location, extra ^210^Pb would more likely signify a local source of pollution. Such a source might be, e.g., a material rich in ^226^Ra and emanating ^222^Rn, industrial processing of ores rich in ^238^U or the burning of coal or other fossil fuels. In our mosses, the activity concentrations of ^210^Pb were 183–690 Bq kg^−1^. As in the case of ^137^Cs, a future measurement of the activity concentration of ^210^Pb above the mentioned values could signify a possibility of an ecologically undesirable human activity.

## Data Availability

The datasets used during the current study available from the corresponding author on reasonable request.
